# Is callose required for silicification in plants?

**DOI:** 10.1098/rsbl.2018.0338

**Published:** 2018-10-03

**Authors:** Gea Guerriero, Ian Stokes, Christopher Exley

**Affiliations:** 1 Environmental Research and Innovation Department, Luxembourg Institute of Science and Technology, Esche/Alzette, Luxembourg; 2 The Birchall Centre, Lennard-Jones Laboratories, Keele University, Staffordshire, UK

**Keywords:** biogenic silica, silicic acid, silicification, callose, rice

## Abstract

The cell wall polymer callose catalyses the formation of silica *in vitro* and is heavily implicated in biological silicification in *Equisetum* (horsetail) and *Arabidopsis* (thale cress) *in vivo*. Callose, a β-1,3-glucan, is an ideal partner for silicification, because its amorphous structure and ephemeral nature provide suitable microenvironments to support the condensation of silicic acid into silica. Herein, using scanning electron microscopy, immunohistochemistry and fluorescence, we provide further evidence of the cooperative nature of callose and silica in biological silicification in rice, an important crop plant and known silica accumulator. These new data along with recently published research enable us to propose a model to describe the intracellular events that together determine callose-driven biological silicification.

## 1. Introduction

We have identified the β-1,3 glucan, callose, as a template for silica deposition in horsetail (*Equisetum arvense*) [1]. All of the major sites of silicification in horsetail, including cell walls, cell plates, plasmodesmata, epidermal papillae, stomata and pollen mirror callose deposition. Where callose is integral to horsetail physiology we also observe deposition of biogenic silica. The detail of this relationship is especially evident in the differentiation of stomata, also observed for fern [2].

*Equisetum* is well known as a plant that accumulates silica in its tissues. Although thale cress (*Arabidopsis thaliana*) does not, it can be induced to do so and especially in trichomes, when engineered to over express callose synthase as a response to stress [3]. Silica deposition acts as an integral, if adventitious, part of a callose-mediated response to pathogen attack by forming an impenetrable barrier to fungal hyphae at the outer epidermis [4]. Recent research has confirmed and extended our observations by demonstrating that callose is essential for cell wall silicification of trichomes in thale cress [5]. These authors elegantly demonstrate the dual role of the exocyst, involved in tethering vesicles to plasma membranes, and callose synthase in callose deposition in trichomes, processes that they conclude are indispensable for silicification. This new research supports our contention that the mechanism of callose-mediated silica deposition involves vesicular transport of, probably, a callose-silicic acid precursor [1]. Multivesicular bodies (MVBs) are implicated in loading callose into papillae in barley (*Hordeum vulgare*) under attack by powdery mildew (*Blumeria graminis* f. sp. *hordei*) [6]. Similarly, work on *Vicia faba* infected by cowpea rust fungus shows that the contents of clathrin-coated pits, coated vesicles and MVBs all reacted with monoclonal and polyclonal β-1,3 glucan-specific antibodies [7]. Papillae formed at sites of pathogen infection become silicified and present a physical barrier to fungal penetration [8]. Epidermal papilla-like projections are also silicified and present similar barriers in a number of species, including horsetail [1,4] and rice (*Oryza sativa*) [9].

## 2. New evidence of callose-mediated silica deposition

**Figure 1.**
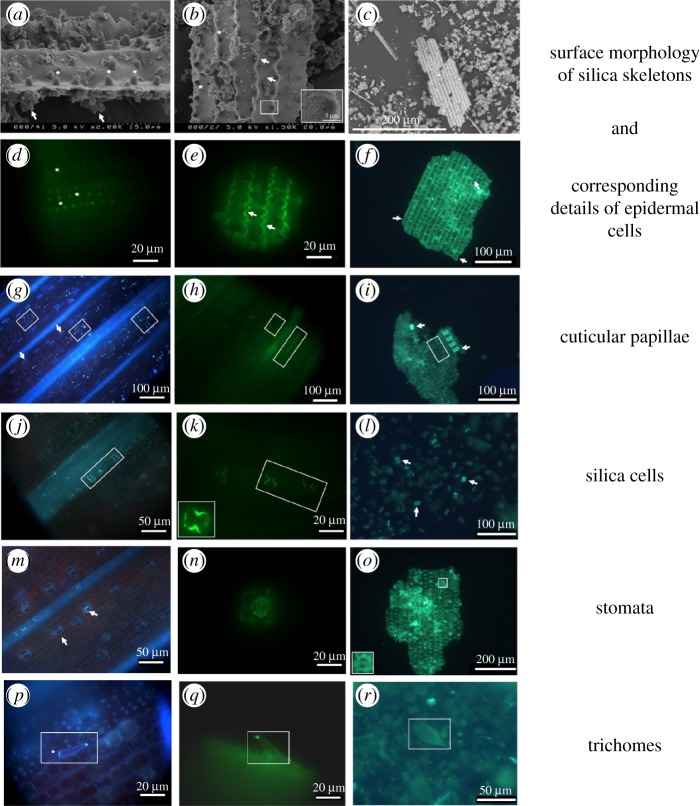
Identification of silica and callose co-occurring in rice leaves by scanning electron microscopy, immunofluorescence and 2-(4-pyridyl)-5-((4-(2-dimethylaminoethylaminocarbamoyl)-methoxy)phenyl)oxazole (PDMPO) fluorescence [1,3,4] (electronic supplementary material, Methods). (*a*) Upper side of silica skeleton showing small cuticular papillae projecting from epidermal cells (asterisks) and granular material accumulating beneath the surface in specific regions (arrows). (*b*) Lower side of silica skeleton showing recesses corresponding to cuticular papillae (asterisks), lobes and necks of epidermal cells (arrows) and porous texture of the silica (boxed region and inset). (*c*) Silica skeleton of epidermal fragment among isolated silica cells showing silicified long cells with undulating walls. (*d*) Callose immunofluorescence highlighting cuticular papillae projecting from the epidermis (asterisks). (*e*) Callose immunofluorescence coinciding with undulating walls of epidermal cells (arrows). (*f*) Silica skeleton showing PDMPO fluorescence associated with epidermal cells (arrows). (*g*) Aniline-blue staining of leaf epidermis showing callose associated with cuticular papillae (boxed regions) and stomata (arrows). (*h*) Callose immunofluorescence of cuticular papillae (boxed regions). (*i*) Silica skeletons revealing strong PDMPO fluorescence of silica cells (arrows) and cuticular papillae (boxed region). (*j*) Aniline-blue staining of leaf epidermis with callose coinciding with silica cells (boxed region). (*k*) Callose immunofluorescence of silica cells (boxed region). Particularly strong is the callose signal in the neck of the silica cell (inset). (*l*) Silica skeletons showing strong PDMPO fluorescence of isolated silica cells (arrows). (*m*) Aniline-blue staining of leaf epidermis identifying callose associated with stomata (arrows). (*n*) Immunofluorescence of callose in a stoma. (*o*) Silica skeletons imaged with PDMPO identifies cuticular papillae, silica cells and a stoma (boxed region and inset). (*p*) Aniline-blue staining of leaf epidermis showing callose at the tip and at what appears to be the Ortmannian ring (asterisk) of a trichome (boxed region). (*q*) Immunofluorescence showing callose at the tip of a trichome (boxed region). (*r*) PDMPO fluorescence of silica skeleton showing silicified trichome (boxed region).

Rice was grown hydroponically in the presence of 1 mM silicic acid and tissue samples obtained and processed according to their subsequent viewing by scanning electron microscopy, fluorescence microscopy and immunofluorescence (see the electronic supplementary material, Materials and Methods). As such, we were able to extend our previous observations on horsetail to this commercially important crop. Herein we show, in a range of leaf cell types in rice, clear co-occurrence of silica and callose (figure 1). This is evident in jigsaw puzzle-like epidermal cells (*e*,*f*), small cuticular papillae (*g*–*i*), silica cells (*j*–*l*), stomata (*m*–*o*) and trichomes (*p*–*r*). Silica skeletons perfectly reproduce the fine details of epidermal cells (*a*–*c* and *f*), while immunofluorescence of intact tissue identifies the presence of callose in papillae and cell walls (*d* and *e*). The callose signal is strongest at the tips of cell lobes (*e*), reminiscent of callose-rich wall subdomains described during the development of mesophyll cells in *Zea mays*, *Vigna sinensis* and *Asplenium nidus* [10,11].

Although callose is identified as a pre-requisite for silica deposition its distribution does not necessarily imply a perfect co-localization with silica at all (sub)cellular levels. Herein the co-occurrence of callose and silica in papillae (*g*–*i*), silica cells (*j*–*l*) and stomata (*m*–*o*) is demonstrated in intact tissues and silica skeletons by aniline-blue staining and immunofluorescence and 2-(4-pyridyl)-5-((4-(2-dimethylaminoethylaminocarbamoyl)-methoxy)phenyl)oxazole (PDMPO) fluorescence respectively. The co-occurrence of callose and silica is striking in trichomes. Aniline-blue stains the trichome tips, as well as a structure that appears to correspond to the Ortmannian ring [12] (*p*); immuno-fluorescence confirms the signal at the tip (*q*) and PDMPO shows silicification of the entire trichome (shaft and tip; *r*).

## 3. A mechanism of callose-mediated silicification

**Figure 2.**
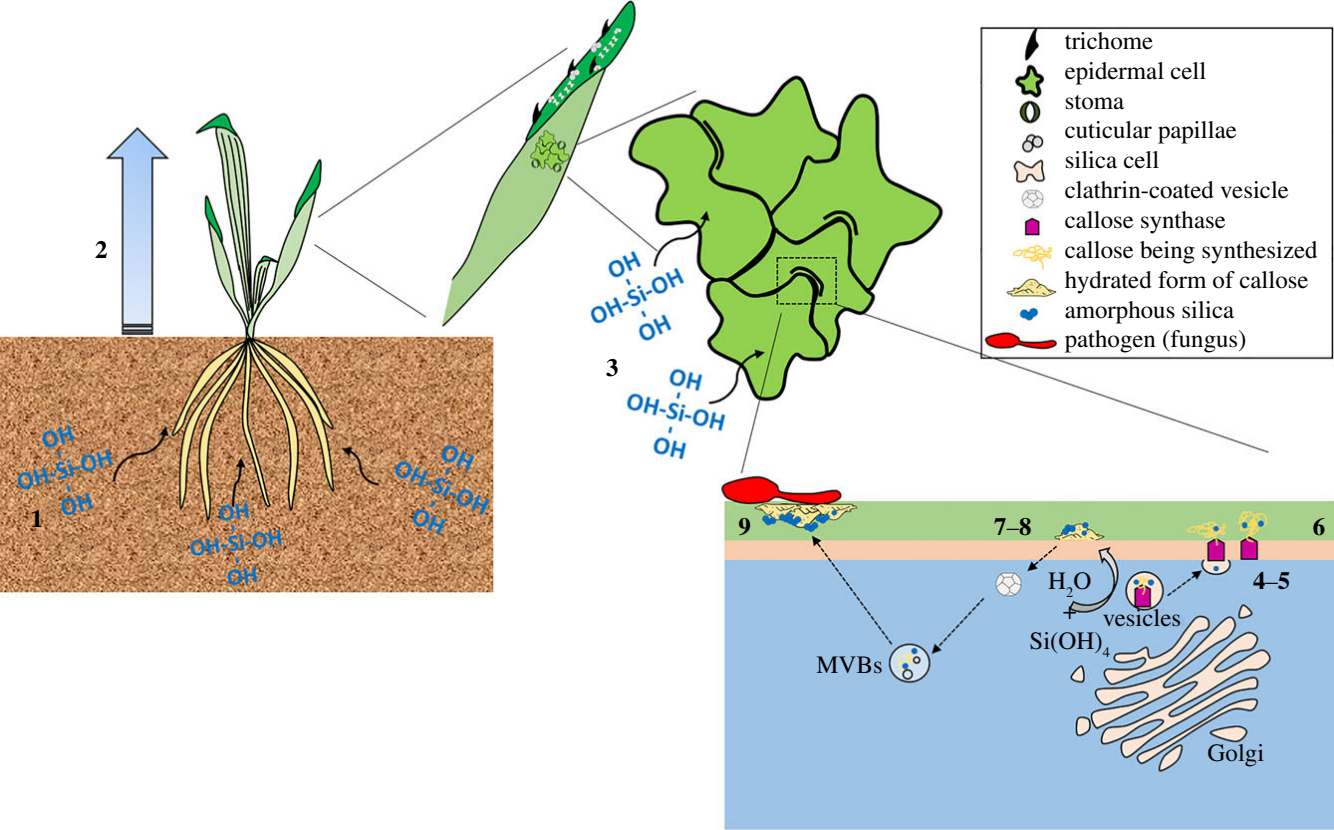
Schematic of proposed mechanism of callose-mediated silicification. **1.** Silicic acid (Si(OH)_4_) follows water from the soil solution into the plant under hydraulic pressure. **2.** Assuming a critical concentration of silicic acid is present in the soil solution, then ‘silicic acid resistors', water channels such as aquaporins, help to sustain a super-saturated concentration of silicic acid within the water conducting channels including xylem. **3.** Silicic acid follows water into and throughout plant tissues. **4.** In the intracellular environment, silicic acid enters (with water) vesicles involved with the transport and use of callose. **5.** In vesicles, both callose and silicic acid adopt a precursor state. Callose may be in a concentrated form (similar to mucin in mucus biochemistry). Silicic acid, while no longer being silicic acid (and so maintaining a concentration gradient of silicic acid from the cell cytosol to the inside of the vesicle) has not yet formed silica. Hydrogen bonding between adjacent hydroxyl groups on silicic acid and callose may support this intermediate structure. A callose synthase in the process of forming callose is depicted, on the basis of the evidence reported by us herein and previously [1,4]. **6.** Callose in vesicles is transported to its point of use and following vesicle tethering to a membrane is secreted along with its polycondensate of silicic acid. **7.** Callose is ‘used' which may involve its further hydration (and implicitly the further import of silicic acid from the cytosol) and an amorphous and malleable form of silica complements the use of callose. **8.** In certain circumstances, for example, in the differentiation of stomata, the ‘use' of callose involves series of steps in which callose is continuously modelled according to requirements, series of steps that might involve both its degradation (β-1,3 glucanases) and synthesis (callose synthases). The intimate association between callose and a silica precursor means that the latter mirrors exactly the steps followed by callose. Callose templates silica ‘deposition' at each stage of its use in plant cell physiology. **9.** Callose is in everyday use throughout plant cell physiology (e.g. response to pathogens). However, additional synthesis, transport and use of callose can be induced as part of a plant's (immune) defence response. Hence, the deposition of silica can be increased in any area of a plant where callose is secreted as part of a defence response. An excellent example of this is in thale cress where increased callose deposition, induced by an elicitor mimicking fungal infection, is coincident with significantly increased deposition of silica in trichomes and mesophyll [3].

The mechanism by which silicon protects against biotic stress remains equivocal [13,14]. It has been reported that both active and passive defence mechanisms are involved in the prophylactic effect of silicon in plants [15]. Herein, we present an argument that in plants callose is integral to silicon's role in protecting against fungal infection. Burgeoning evidence that callose and callose biochemistry are essential for silica deposition in plants now argues that silicification is a process that begins in the intracellular environment (figure 2). It is neither dependent upon the transpiration stream, nor evaporation of water at leaves nor other external surfaces. Indeed, in support of such a contention, it was recently shown that active processes, rather than passive transpiration, are involved in silicification in *Sorghum* silica cells [16]. Current evidence supports the fact that some plants are capable of maintaining a super-saturated concentration of silicic acid in their xylem [2] with the proviso that the concentration of silicic acid in their soil water is above a critical threshold [4]. Silicic acid in xylem and other water-conducting vessels will follow water into the tissues down a concentration gradient. This concentration gradient into the cell cytosol will be maintained by the capture or harvesting of silicic acid into exocytic vesicles and MVBs involved in the transport and use of callose [7,17]. The degree to which silica mirrors the use of callose in horsetail [1], fern [2] and rice points forcibly towards their co-localization in vesicles. If so, this co-localisation does not affect the role or function of callose and it is also unlikely that it catalyses the precipitation of silica within the transport vesicle. It is more likely that both exist as precursors to the form in which they will eventually be used (callose) and deposited (silica). Perhaps callose is transported in vesicles as a concentrate and is only fully hydrated, as required by its immediate function, *in situ* upon its secretion? An interesting analogy would be mucin, the glycopeptide that is the precursor to mucus, which only upon its secretion is hydrated as per its functional requirement. Such a mechanism for callose should fit the myriad applications of this amorphous polysaccharide in plant cell physiology (examples including, cell division, pollen tube growth, wound and pathogen response, microsporogenesis, stomata closure). Concomitant with the hydration of secreted callose will be the import of silicic acid from the cytosol and this additional silicic acid might be the trigger for further polymerization and condensation of the secreted vesicular silicic acid condensate. Many of the roles of callose are ephemeral and their temporary nature does appear to be mimicked in some instances by silica, this being especially evident in the role played by callose in the differentiation of stomata [1]. This means that when callose is unravelled/metabolized, silica is likewise, which strongly suggests that their co-localization supports the existence of a form of hydrated silica which can be modelled, dissolved and formed as the conditions of the immediate vicinity dictate. The use of callose is remarkably mirrored by silica deposition. However, callose should not necessarily be considered as a final marker for silicification only, perhaps, a mode of delivery of silica towards a final destination. For example, cell walls are heavily silicified in horsetail, but probably cellulose *per se* does not mediate this process, it perhaps like other cell wall components such as mixed-linkage glucans or lignin [18], acts only as a compartment in accepting the ripening silicic acid condensate as it forms amorphous hydrated silica.

## 4. Future research

Future research should both develop understanding of the mechanism of callose-mediated silicification and, importantly, look to translate these advances into non-invasive methods of crop protection. For example, current use of fungicides against powdery mildew in valuable crops could be replaced by a strategy of callose-induced silica deposition in epidermal tissues. Additional accrued benefits of enhanced silica deposition could include increased capture of carbon dioxide [19], reflecting the close association that exists between silicic acid and the global climate [20].

## Supplementary Material

Materials and Methods;Figure to promote the paper

## Data accessibility

This article has no additional data.
