# Supplementary material for: Is callose required for silicification in plants?
Source: Biol Lett. 2018 Oct 3;14(10):20180338. doi: 10.1098/rsbl.2018.0338 (PMC6227863; doi:10.1098/rsbl.2018.0338)
Supplement: Materials and Methods;Figure to promote the paper [file rsbl20180338supp1.docx]

**Materials and Methods**

*Hydroponic Culture*

Rice seeds (*Oryza sativa* Koshihikari) were germinated on filter papers in Petri dishes and thereafter grown hydroponically in 1/5^th^ Murashige & Skoog nutrient solution supplemented with silicon (Na_4_SiO_4_ Alfa Aesar) to give a 1mM concentration of silicic acid. Nutrient solutions were replaced every 4 days and plants were grown under grow lights delivering 4,450 Lumens using a 12:12 day:night regime.

*Preparation of Plant Tissues for PDMPO Fluorescence and SEM*

Rice plant samples were separated according to their anatomical region and cut with small scissors to a length of 1cm. The samples were digested in PFA Teflon© vessels (<0.5g) with venting plugs and screw caps (CEM Microwave Technology Ltd, UK) using a 1:1 mixture of 15.8M HNO_3_ and 18.4M H_2_SO_4_. Vessels were placed in insulating sleeves on a turntable, capable of holding up to 40 vessels. The microwave digestion programme was set up with Mars Xpress Microwave (CEM Microwave Technology Ltd, UK) using a CEM provided Tissue Express organics method.

Digested samples were diluted with ultrapure water (cond. <0.067μS/cm) and silica was collected by filtration (Whatman 0.45µm filter paper) using several volumes of ultrapure water to rinse and clean the silica samples. Filter papers were placed in Petri dishes in an incubator to dry.

*PDMPO Fluorescence Microscopy*

Silica was immersed in 20mM PIPES buffer at pH7 adjusted with dilute NaOH (Acros Organics, Mw 302.35) with 0.125µM PDMPO (LysoSensor Yellow/blue DND-160 1mM in DMSO). After 24h incubation 50µL of the silica/PDMPO preparation was added to a cavity slide, covered with a cover slip and viewed using an Olympus BX50 fitted with a BXFLA fluorescent attachment using a U-MWU filter cube (Ex: 333-385nm; Em: 400-700 nm). A ColourView III digital camera (OSIS FireWire Camera 3.0 digitizer) was used to capture

images in conjunction with CELL* Imaging software (Olympus Cell* family, Olympus Soft Imaging solutions GmbH 3.0).

*Callose Immunfluorescence*

Identification of callose by immunofluorescence and fluorescence microscopy was carried out according to an established method briefly described herein. Small sections of rice tissues (<1mm thickness) were cut by hand with a scalpel, fixed and the cellulose in cell walls digested using 1% cellulase (Onazuka R-10, Yakult Pharm. Japan). Callose detection was performed on the extracted digested tissue using a (1-3)-β-glucan antibody (1:40; Biosupplies) and a secondary anti-mouse IgG-FITC antibody (1:40). Finally we used a Hoechst 33258 DNA counterstain and samples were mounted on glass slides and cover slipped. Tissue sections were viewed with a Zeiss Axioplan microscope (Blue Filter Cube #487910; Ex: 450-490nm; Em: 515-565 nm) and images were captured using a Zeiss Axiocam MRc5 digital camera.

*Scanning Electron Microscopy*

Silica skeletons were mounted onto 12mm sticky back carbon tabs (Agar Scientific) fixed to SEM stubs and viewed using either a Hitachi TM3000 Microscope or a Hitachi S-4500 SEM. For the latter samples were gold-plated prior to viewing.
